# An Investigation into the Cytotoxic Effects of 13-Acetoxysarcocrassolide from the Soft Coral *Sarcophyton crassocaule* on Bladder Cancer Cells 

**DOI:** 10.3390/md9122622

**Published:** 2011-12-13

**Authors:** Ching-Chyuan Su, Jui-Hsin Su, Jen-Jie Lin, Cheng-Chi Chen, Wen-Ing Hwang, Han Hsiang Huang, Yu-Jen Wu

**Affiliations:** 1 Antai Medical Care Cooperation Antai Tian-Sheng Memorial Hospital, Pingtung 92842, Taiwan; Email: a081001@mail.tsmh.org.tw; 2 National Museum of Marine Biology and Aquarium, Pingtung 94446, Taiwan; Email: x2219@nmmba.gov.tw; 3 Department of Beauty Science, Meiho University, Pingtung 91202, Taiwan; Email: q87634@yahoo.com.tw (J.-J.L.); x00002073@meiho.edu.tw (C.-C.C.); 4 Department of Food Science and Nutrition, Meiho University, Pingtung 91202, Taiwan; Email: x00000018@meiho.edu.tw

**Keywords:** 13-acetoxysarcocrassolide, *Sarcophyton crassocaule*, BFTC cells, proteomic analysis

## Abstract

Active compounds from natural products have been widely studied. The anti-tumor effects of 13-acetoxysarcocrassolide isolated from Formosan soft coral *Sarcophyton crassocaule* on bladder cancer cells were examined in this study. An MTT assay showed that 13-acetoxysarcocrassolide was cytotoxic to bladder female transitional cancer (BFTC) cells. We determined that the BFTC cells underwent cell death through apoptosis by flow cytometry. Due to the highly-migratory nature of the BFTC cells, the ability of 13-acetoxysarcocrassolide to stop their migration was assessed by a wound healing assay. To determine which proteins were affected in the BFTC cells upon treatment, a comparative proteomic analysis was performed. By LC-MS/MS analysis, we identified that 19 proteins were up-regulated and eight were down-regulated. Seven of the proteins were confirmed by western blotting analysis. This study reveals clues to the potential mechanism of the cytotoxic effects of 13-acetoxysarcocrassolide on BFTC cells. Moreover, it suggests that PPT1 and hnRNP F could be new biomarkers for bladder cancer. The results of this study are also helpful for the diagnosis, progression monitoring and therapeutic strategies of transitional cell tumors.

## 1. Introduction

Urothelial carcinomas are the fourth most common tumors after prostate (or breast) cancer, lung cancer, and colorectal cancer. Bladder tumors account for 90–95% of urothelial carcinomas. It has been estimated that approximately 90% of human bladder tumors are present as transitional cell tumors [1,2]. Transitional cell tumors have high incidence in adults and are a common cause of death of genitourinary tumors [3,4], which explains why transitional cell carcinoma in the elderly population is becoming increasingly important [5]. It has also been reported that the percentage of female patients with cancer rises with increasing age [6]. Typical characteristics of transitional cell tumors are their multifocal origin and high recurrence-rate (about 60%–70%) after transurethral resection. Some agents like mitomycin C and adriamycin have been used for intravesical chemotherapy. This treatment has diminished the early recurrence-rate in about 14% of patients. Intravesical bacille Calmette-Guérin (BCG) has been reported to have the greatest effects with the highest response rate on transitional cell carcinoma despite its frequent side effects [7,8]. It is essential to find new diagnosis and treatment measures for superficial transitional cell carcinoma. 

**Figure 1:**
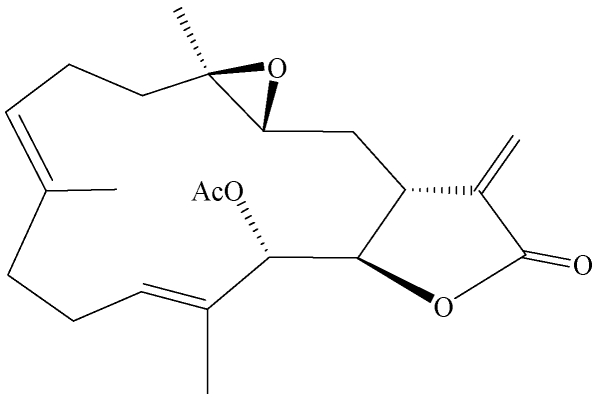
Chemical structure of 13-acetoxysarcocrassolide.

The therapeutic applications of natural products isolated from soft corals have been widely investigated [9,10,11]. Several steroid compounds such as diterphenoids, diterpenes, and prostanoids have been isolated from marine soft corals. These types of compounds have been reported to induce apoptosis and are cytotoxic against different cancer cell lines, such as prostate, breast, colon, cervical, liver, and oral cancer cell lines [12,13,14,15,16,17]. Three diterpenoids, 1-*epi*-leptocladolide A, leptocladolides A, and 7*E*-leptocladolide A isolated from the Taiwanese soft coral *Sinularia parva* were found to have significant cytotoxic effects against KB and Hepa59T/VGH cancer lines [18]. Reports on activities of compounds from natural products on human bladder cancer cells are very limited. Bladder female transitional cancer (BFTC) cells have been widely used in biomedical and urological studies of bladder tumors [4,19,20]. In previous studies a series of novel secondary metabolites, including cembranes [21,22,23,24,25,26,27,28,29,30,31,32,33], steroids [22,28,30], hippurins [31], prostaglandins [32] and others [33] have been isolated from the Formosan soft coral *Sarcophyton crassocaule*. Some of these have been found to possess several kinds of biological activities, such as cytotoxic [22,24,25,27,28] and anti-inflammatory activity [27,28]. In the current study, the cytotoxic effects of a cembrenolide diterpene, 13-acetoxysarcocrassolide (Figure 1), isolated from *Sarcophyton crassocaule* on BFTC cells have been examined. Proteomic and western blotting analysis was carried out to investigate and confirm the changes of protein expression in BFTC cells after 13-acetoxysarcocrassolide treatment. The data in this study provide information for understanding the biochemical aspects of the cytotoxic effects of 13-acetoxysarcocrassolide on BFTC cells and will help to develop tools for diagnosis and progression monitoring of transitional cell tumors.

## 2 Materials and Methods

### 2.1. Materials

Cell Extraction Buffer was obtained from BioSource International (Camarillo, CA, USA). Protease inhibitor cocktail was from Sigma (St Louis, MO, USA). The IPG buffer (pH 4–7) for two-dimensional gel electrophoresis (2-DE) was purchased from GE Healthcare (Buckinghamshire, UK). Rabbit anti-human heat shock protein 60 (HSP60), isocitrate dehydrogenase (IDH), Stress-70 protein, heat shock cognate 71 kDa protein (HSC71), heterogeneous nuclear ribonucleoproteins F (hnRNP F), heterogeneous nuclear ribonucleoproteins C1/C2 (hnRNP C1/C2) and protein disulfide-isomerase A3 (PDIA3) antibodies were obtained from ProteinTech Group (Chicago, IL, USA). Antibodies against caspase-3, cleaved caspase-3, caspase-8, caspase-9, cleaved caspase-9 and Bcl-xL were from Cell Signaling Technology (Danvers, MA, USA). Antibodies against cytochrome C and p53 were from Epitomics (Burlingame, CA, USA). Rabbit anti-human β-actin antibodies were obtained from Sigma. Goat anti-rabbit and horseradish peroxidase conjugated IgG was from Millipore (Bellerica, MA, USA). PVDF (Polyvinylidene difluoride) membranes and Chemiliminescent HRP Substrate were from Pierce (Rockford, IL, USA). 

### 2.2. Cell Culture and the Treatment with 13-Acetoxysarcocrassolide

BFTC cells were cultured in DMEM with 4 mM l-glutamine adjusted to contain 1.5 g/L sodium bicarbonate and 4.5 g/L glucose, supplemented with 10% (v/v) FBS, 100 units/mL penicillin, 100 μg/mL streptomycin, 1 mM sodium pyruvate, and 0.01 mg/mL human transferrin in a humidified incubator with 5% CO_2_ at 37 °C.When cells were above 70% confluent, subculture was conducted at a split ratio of 1:6. The isolation of 13-acetoxysarcocrassolide from the soft coral Sinularia *leptoclado* was accomplished according to the reported procedures [34]. Control cultures were prepared by adding DMSO at the same final concentration as in the treated samples (0.01% v/v). DMSO was used to dissolve 13-acetoxysarcocrassolide. BFTC cells were treated with different concentrations of 13-acetoxysarcocrassolide (0.5 μg/mL, 1.0 μg/mL, 1.5 μg/mL, 3.0 μg/mL and 5.0 μg/mL) and harvested after incubation for 24 h. All the experiments were repeated three times.

### 2.3. MTT Assay

The anti-proliferative effect of 13-acetoxysarcocrassolide on BFTC cells was measured by MTT assay. BFTC cells were seeded at a density of 1 × 10^5^/cm^2^ in 96 well plates. After the addition of 0.5–5.0 μg/mL 13-acetoxysarcocrassolide for 24 h, the MTT solution (1 mg/mL in PBS) was added to each well. The plates were then incubated at 37 °C for 4 h. DMSO was applied to each well to achieve solubility of purple-blue MTT formazan crystals in viable cells. The optimal density (O.D) was measured at 595 nm by a microplate ELISA reader with DMSO as the blank. Data were presented as ±standard error of mean (SEM) of the pooled data. Statistical comparisons of two or more groups of data were carried out using one-way analysis of variance (ANOVA) followed by the Tukey-Kramer multiple comparison test to determine the significant differences between the experimental groups by GraphPad Instat statistical software (San Diego, CA, USA) [35].

### 2.4. Wound-Healing Assay

The anti-migratory and anti-motility effects of 13-acetoxysarcocrassolide on BFTC cells were examined by a wound healing assay. BFTC cells were seeded in 6 well plates 18 h before the addition of 13-acetoxysarcocrassolide. An artificial wound was created with a 10 μL pipette tip at 0 h. Unattached tumor cells were removed by the wash with PBS. Images of the experimental groups (0 μg/mL, 1.0 μg/mL, 1.5 μg/mL and 3.0 μg/mL 13-acetoxysarcocrassolide) were acquired at 0 h, 6 h, 12 h and 24 h after the treatment of 13-acetoxysarcocrassolide. The images of migrated tumors cells inside the wound were acquired by an inverted light microscope with an imaging system (Nikon).

### 2.5. Analysis of Cell Cycle and Apoptosis

Harvested BFTC cells were fixed in ice-cold 70% ethanol at 4 °C for 30 min. After centrifugation, the cell pellets were washed and resuspended in PBS. Tumor cells were then treated with ribonuclease A (25 mg/L) and 0.5% Triton X-100 at 37 °C for 60 min. Cellular DNA was stained with propidium iodide (50 mg/L) for 30 min. After centrifugation, the pellets were resuspended in PBS. The cellular DNA in 104 cells was analyzed in Elite-ESP flow cytometer (Beckman Coulter, Miami, FL, USA) and fixed with an argon laser set at 488 nm. The percentage of apoptosis was determined by Elite-ESP software program (Beckman Coulter, Miami, FL, USA). 

### 2.6. Protein Extraction and Preparation

BFTC cells treated with 0, 0.5, 1.0, 1.5, 3.0 and 5.0 μg/mL 13-acetoxysarcocrassolide for 24 h were lysed with Cell Extraction Buffer (BioSource International) and protease inhibitor cocktail (Sigma). The total protein in the supernatant was then precipitated out by triple the volume of 10% TCA/Acetone solution containing 20 mM DTT at −20 °C overnight. The mixture was then centrifuged at 8000 rpm for 30 min at 4 °C. The supernatant was discarded. The pellet was suspended in rehydration buffer (6 M urea, 2 M thiourea, 0.5% CHAPS, 0.5% IPG buffer, 20 mM DTT, and 0.002% bromophenol blue) at 4 °C for overnight. The protein contents were determined using 2-D Quant Kit (GE Healthcare).

### 2.7. Two-Dimensional Gel Electrophoresis

The first dimension electrophoresis (isoelectric focusing) was performed with GE Healthcare Ettan IPGphor 3 using the reported procedure [36]. Proteins (50 μg) extracted from whole cell were loaded on 11-cm strip. Every 11-cm IPG strip (pI 4–7, Immobiline DryStrip) was rehydrated at 30 V for 12 h and then focused according to the preset program: 200 V (2 h), 500 V (2 h), 1000 V (2 h), 4000 V (1 h), 8000 V (4 h), until the total Vh reached 39,400. The equilibrated strip was placed on the top of a SDS-PAGE gel (12.5%) and then the second dimension electrophoresis was run at 150 V for 6.5 h. The 2-DE gels were stained with silver staining and then subjected to image analysis with the PDQuest 2-D software (version 7.1.1). 2-DE images were taken in triplicate for each sample and normalized prior to statistical analysis. 

### 2.8. Protein Identification by LC-MS/MS

The protein spots of interest were excised, destained and then subjected to tryptic in-gel digestion as described in a previous report [37]. MS analysis was performed using a AB SCIEX QTRAP^®^ 5500Q mass spectrometer (Applied Biosystems, CA, USA). A peptide mixture was separated by nanoflow reversed phase C18 chromatography on nano LC using the Agilent 1200 System and PepMap100 C18, 75 μm × 15 cm (3 μm) nanoLC column or HPLC using the Agilent NanoLC 1200 System and Agilent Zobax 2.1 mm × 150 mm C18 column. LC-MS/MS analysis employed a 10-min online trapping and desalting step followed by a 60-min 5–40% mobile phase B gradient at nano flow and a 15-min 5–40% mobile B phase gradient at higher flow (mobile phase B = 98% ACN, 0.1% formic acid). The scan range was from *m/z* 100 to 1000 for MS. The raw data was processed into a text file format of WIFF with Analyst 1.5.1. MASCOT was used in searching for protein identification by NCBInr protein database.

### 2.9. Western Blot Analysis

Western blotting was conducted to verify the regulation of 7 differential 2-DE-detected proteins and changes of the pro-apoptotic factors correlated with HSP60 and hnRNP F. The protein samples under reducing conditions were separated by 10% SDS-polyacrylamide gel electrophoresis (SDS-PAGE). The proteins on the gel were then transferred to PVDF membranes. The membranes were incubated with rabbit polyclonal antibodies against human HSP60, stress-70 protein, HSC71, IDH, hnRNP C1/C2, hnRNP F, PDIA3, caspase-3, cleaved caspase-3, caspase-8, caspase-9, cleaved caspase-9, p53, cytochrome C, Bcl-xL and β-actin at 4 °C for 2 h or overnight. The membranes were washed three times in PBST (10 mM NaH_2_PO_4_, 130 mM NaCl, 0.05% Tween 20), then incubated with the second antibodies (goat anti-rabbit and horseradish peroxidase conjugate, 1:5000 in blocking solution) for 1 h. After washing with PBST for three times, the blots were visualized through chemiluminesence by adding ECL western blotting reagents on the Alpha Innotech System (San Leandro, CA, USA).

## 3 Results

### 3.1. The Anti-Proliferative Effect of 13-Acetoxysarcocrassolide on BFTC Cells

**Figure 2:**
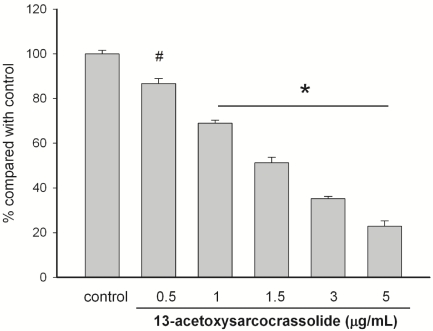
Viability of bladder female transitional cancer (BFTC) cells exposed to increasing concentrations of 13-acetoxysarcocrassolide. 13-acetoxysarcocrassolide dose-dependently suppressed the growth of BFTC cells (^#^ *P* < 0.01 and * *P* < 0.001). Inhibitory effects on cell proliferation were assessed by MTT assay as described in Materials and Methods. Data were presented with the mean ± SEM from three independent experiments.

**Figure 3:**
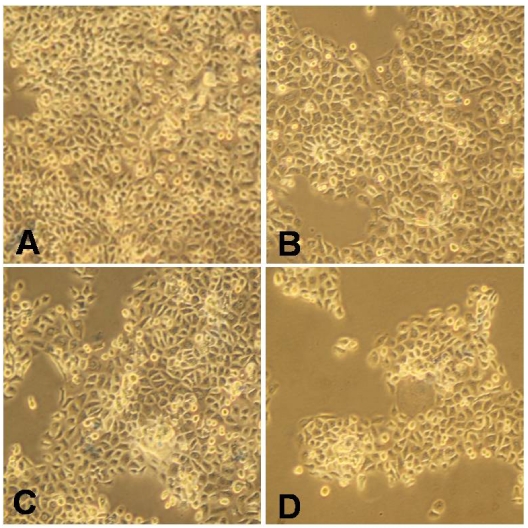
Decreased population and morphological changes of BFTC cells treated with different concentrations of 13-acetoxysarcocrassolide for 24 h. (**A**) control; (**B**) 1 μg/mL; (**C**) 1.5 μg/mL; and (**D**) 3 μg/mL of 13-acetoxysarcocrassolide.

The anti-proliferative effect of 13-acetoxysarcocrassolide was measured by the colorimetric MTT assay. The concentration of 13-acetoxysarcocrassolide ranging from 0.5 μg/mL to 5 μg/mL dose-dependently inhibited the growth of BFTC cells (*P* < 0.01 and *P* < 0.001, Figure 2). Under the observation of inverted light microscopy, the population of BFTC cells was clearly reduced after treatment with 1.5 and 3 μg/mL (Figure 3).

### 3.2. The Anti-Migratory Effect of 13-Acetoxysarcocrassolide on BFTC Cells

**Figure 4:**
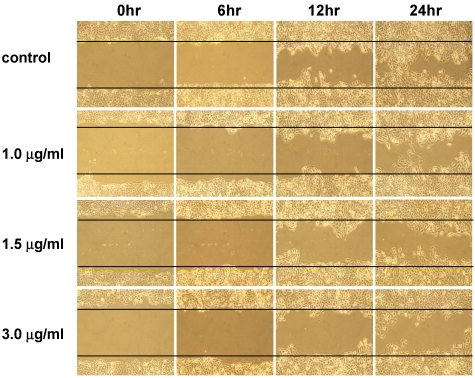
Wound healing assay of BFTC cells treated with increasing concentrations of 13-acetoxysarcocrassolide. Cells in the areas between the two solid lines were those migrated in the gaps during the indicated time periods. It was seen that increased 13-acetoxysarcocrassolide concentration decreased the migration ability of BFTC cells.

The anti-migratory effect of 13-acetoxysarcocrassolide was examined using a wound healing assay. The decreased areas of artificial wounds were observed after treatment with 13-acetoxysarcocrassolide. BFTC cell migration was reduced with increasing concentrations and exposure time of 13-acetoxysarcocrassolide. The experiments were repeated three times and migrated BFTC cells inside the wound were significantly reduced at 12 and 24 h after exposure to 1, 1.5 and 3 μg 13-acetoxysarcocrassolide (* *P* < 0.001) (Figure 4 and Table 1). 

**Table 1:** BFTC cells were exposed to increasing concentrations of 13-acetoxysarcocrassolide. Migrated BFTC cells inside the wound were counted after the indicated time periods. The data of cell count are from three individual assays and analyzed using ANOVA followed by the Tukey-Kramer multiple comparisons test and presented as mean ± SEM (* *P* < 0.001).

	0 h	6 h	12 h	24 h
control	8.333 ± 3.215	46 ± 8	158.67 ± 18.175	344 ± 14.177
1 μg 13-acetoxysarcocrassolide	10.67 ± 3.512	17.67 ± 6.506	107.31 ± 5.275 *	219.67 ± 21.197 *
1.5 μg 13-acetoxysarcocrassolide	5.33 ± 2.309	14 ± 5	79 ± 10.44 *	155.33 ± 12.22 *
3 μg 13-acetoxysarcocrassolide	4.67 ± 3.055	11.33 ± 5.508	53.33 ± 15.503 *	105.33 ± 12.662 *

### 3.3. Apoptosis-Induced Effects of 13-Acetoxysarcocrassolide on BFTC Cells

**Figure 5:**
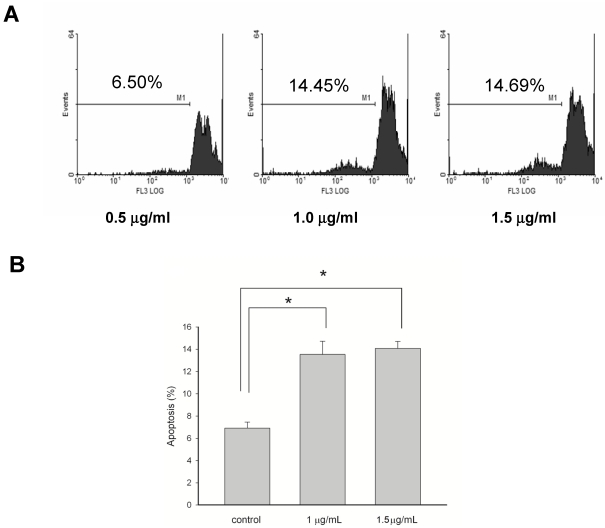
(**A**) Flow cytometric analysis represented by PI staining for the apoptosis-induced effects of 13-acetoxysarcocrassolide on BFTC cells. BFTC cells were exposed to 13-acetoxysarcocrassolide at 0, 1 and 1.5 μg/mL for 24 h, and then collected and stained as described in Materials and Methods. Cells at sub-G1 phase were assessed as apoptotic cells; (**B**) 13-acetoxysarcocrassolide at 1 and 1.5 μg/mL doubled the apoptotic rate compared with that of the control (* *P* < 0.001). Data were pooled from three independent runs and analyzed by ANOVA followed by the Tukey-Kramer multiple comparison test.

The BFTC cells with or without 13-acetoxysarcocrassolide treatment were harvested and stained with propidium iodine. Tumor cells marked at sub-G1 phase were measured as apoptotic BFTC cells. The apoptotic rate of BFTC cells was doubled by the treatment of either 1 or 1.5 μg/mL of 13-acetoxysarcocrassolide in comparison with the control (* *P* < 0.001, Figure 5). There is no statistical difference in the apoptotic rate between the effects of the treatments of 1 and 1.5 μg/mL 13-acetoxysarcocrassolide (Figure 5).

### 3.4. Proteomic Analysis of BFTC Cells Treated with 13-Acetoxysarcocrassolide

**Figure 6:**
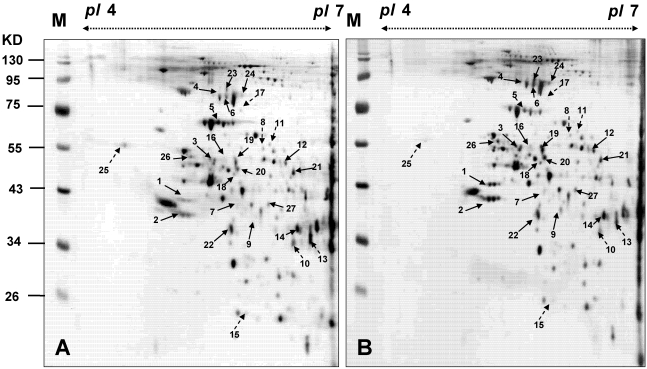
The 2-DE maps of BFTC cells treated with 13-acetoxysarcocrassolide and the control sample. (**A**) control; (**B**) treated with 1.5 μg/mL 13-acetoxysarcocrassolide for 24 h. Proteins spots marked on the maps were considered differentially expressed and identified by LC-MS/MS. The results are representative of three independent runs.

BFTC cells were harvested after being treated with 1.5 μg/mL 13-acetoxysarcocrassolide. The proteins from the cells were extracted and the supernatants were collected. Proteins were precipitated by TCA/Acetone. The 2-DE maps of BFTC cells treated with 13-acetoxysarcocrassolide were compared with those of the control to analyze the effect of 13-acetoxysarcocrassolide on BFTC cells. The 2-DE was completed with a loading of 50 μg protein (pI 4–7) and visualized by silver staining (Figure 6). PDQuest image analysis software (Bio-Rad, CA, USA) was employed to detect the differential protein spots which were defined as the proteins showing a more than 1.5 fold intensity difference in 2-DE maps between the treated BFTC cells and the control. The protein identification was carried out by LC-MS/MS analysis after in gel digestion. MASCOT protein identification search software was used for the identification of the differential protein spots. A total of 27 differential protein spots were successfully identified. A list of the identified proteins with their MASCOT score, MS/MS matched sequences, apparent and theoretical MW, pI, coverage, and folds of change in expression level (up-regulation or down-regulation) are shown in Table 2. 

**Table 2:** A total of 27 differential proteins and their changes identified by LC-MS/MS after treatment with 1.5 μg/mL 13-acetoxysarcocrassolide for 24 h.

Spot no.	Protein name	Accession no.	Calculate Mr/pI	Peptide matched	Sequence covered %	MASCOT score	Regulation (fold-change) *
1	Heterogeneous nuclear ribonucleoproteins C1/C2	P07910	33.65/4.95	10	21	99	+7.0
2	Heterogeneous nuclear ribonucleoproteins C1/C2	P07910	33.65/4.95	14	35	163	+8.3
3	Heterogeneous nuclear ribonucleoprotein F	P52597	45.64/5.38	20	31	201	+2.0
4	Heat shock cognate 71 kDa protein	P11142	70.85/5.37	7	14	93	+3.7
5	60 kDa heat shock protein	P10809	61.01/5.7	35	39	686	−2.0
6	Stress-70 protein	P38646	73.63/5.87	27	37	333	+2.2
7	Arginase-2	P78540	38.55/6.0	2	8	70	+2.0
8	Protein disulfide-isomerase A3 precursor	P30101	54.74/5.98	26	41	387	−2.3
9	l-lactate dehydrogenase B chain	P07195	36.61/5.71	7	16	63	+2.6
10	Isocitrate dehydrogenase [NAD] subunit alpha	P50213	39.56/6.47	7	15	127	−3.2
11	Protein disulfide-isomerase A3 precursor	P30101	54.74/5.98	14	29	211	−1.8
12	Mitochondrial-processing peptidase subunit beta	O75439	54.33/6.38	11	23	126	+2.8
13	Heterogeneous nuclear ribonucleoprotein H3 (hnRNP H3)	P31942	36.9/6.37	3	10	74	−2.1
14	Palmitoyl-protein thioesterase 1 precursor	P50897	34.17/6.07	5	10	83	+1.8
15	Proteasome subunit beta type 4 precursor	P28070	29.18/5.72	6	24	92	−3.0
16	Thioredoxin domain-containing protein 5 precursor	Q8NBS9	47.59/5.63	4	11	69	+2.2
17	Dihydrolipoyllysine-residue acetyltransferase component of pyruvate dehydrogenase complex	P10515	65.73/5.79	5	2	63	−2.0
18	Protein NDRG1	Q92597	42.8/5.49	9	25	129	+2.3
19	Ubiquinol-cytochrome-c reductase complex core protein 1	P31930	52.61/5.94	9	15	156	+2.3
20	Ubiquinol-cytochrome-c reductase complex core protein 1	P31930	52.61/5.94	2	3	67	+2.2
21	Ornithine aminotransferase	P04181	48.5/6.57	23	30	296	+1.5
22	Guanine nucleotide-binding protein G(I)/G(S)/G(T)	P62873	37.35/5.6	12	20	151	+1.9
23	Stress-70 protein	P38646	73.63/5.87	17	21	190	+2.7
24	Heat shock cognate 71 kDa protein	P11142	70.85/5.37	14	17	137	+2.2
25	Calreticulin precursor	P27797	48.11/4.29	13	17	99	−3.2
26	ATP synthase subunit beta	P06576	56.52/5.26	20	34	256	+2.8
27	Glial fibrillary acidic protein	P14136	49.85/5.42	3	4	53	+2.4

* Regulations (fold-changes) of differentially expression proteins are expressed at 24 h treatment of 13-acetoxysarcocrassolide.

The varied expression of proteins distributed throughout the entire gels indicated that multiple clusters of proteins were involved in the effects of 13-acetoxysarcocrassolide on BFTC cells. A total of 19 differential proteins were up-regulated after 13-acetoxysarcocrassolide treatment. These proteins were heterogeneous nuclear ribonucleoproteins C1/C2, heterogeneous nuclear ribonucleoprotein F, heat shock cognate 71 kDa protein, stress-70 protein, l-lactate dehydrogenase B chain, mitochondrial-processing peptidase subunit beta, palmitoyl-protein thioesterase 1 precursor, thioredoxin domain-containing protein 5 precursor, protein NDRG1, ubiquinol-cytochrome-c reductase complex core protein 1, ornithine aminotransferase, guanine nucleotide-binding protein G(I)/G(S)/G(T), ATP synthase subunit beta, and glial fibrillary acidic protein. In contrast, eight down-regulated proteins were 60 kDa heat shock protein, protein disulfide-isomerase A3 precursor, isocitrate dehydrogenase [NAD] subunit alpha, heterogeneous nuclear ribonucleoprotein H3, proteasome subunit beta type 4 precursor, dihydrolipoyllysine-residue acetyltransferase component of pyruvate dehydrogenase complex, and calreticulin precursor. 

### 3.5. Western Blot Analysis

**Figure 7:**
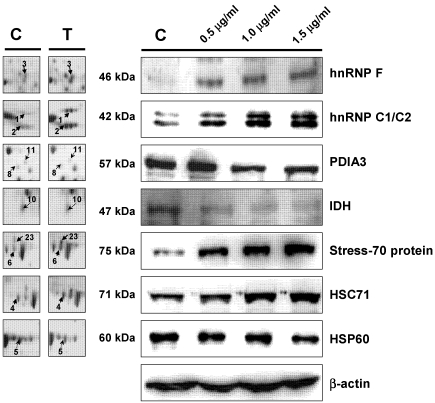
Western blotting analysis of HSP60, stress 70 protein, HSC71, PDIA3, hnRNP F, hnRNP C1/C2, and IDH in BFTC cells treated with increasing concentrations of 13-acetoxysarcocrassolide for 24 h. β-actin was used as the internal control.

**Figure 8:**
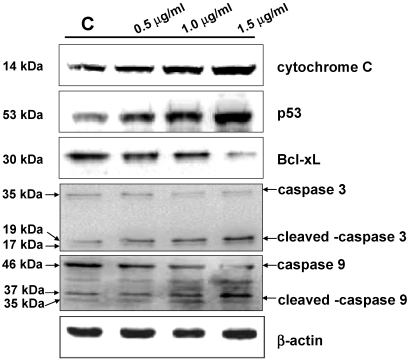
Western blotting analysis of caspase-3, cleaved caspase-3, caspase-9, cleavedcaspase-9, cytochrome C, p53 and Bcl-xL in BFTC cells treated with increasing concentrations of 13-acetoxysarcocrassolide for 24 h. β-actin was used as the internal control.

The varied expression of HSP60, Stress-70 protein, HSC71, hnRNP C1/C2, hnRNP F, PDIA3, and IDH was further verified by western blotting analysis (Figure 7). The results were in accordance with the 2-DE data. Modulation of caspase-3 and caspase-9 after the treatment in BFTC cells was also determined by western blotting analysis. The elevated expression of cytochrome C, p53, cleaved caspase-3, cleaved caspase-9 and a decrease of caspase-3, caspase-9, Bcl-xL in BFTC cells with 13-acetoxysarcocrassolide treatment were observed (Figure 8). Cleaved caspase-8 was not detected in 13-acetoxysarcocrassolide-treated cells and the control (data not shown). Each western blotting analysis is repeated three times and a representative result was exhibited.

## 4. Discussion

In the current study, the cembranoid 13-acetoxysarcocrassolide, isolated from the Formosan soft coral *Sarcophyton crassocaule* exerted anti-proliferation, anti-migration and apoptosis-induced effects on bladder female transitional cancer (BFTC) cells *in vitro*. A comparative proteomic analysis was then conducted by 2-DE, LC-MS/MS and western blotting analysis to determine the potential biomarkers and investigate the induction of cell death in BFTC cells. A total of 27 differential proteins have been identified by LC-MS/MS analysis, including 19 up-regulated and 8 down-regulated proteins in BFTC cells after exposure to 13-acetoxysarcocrassolide. A Western blot analysis confirmed the 2-DE data of HSP60, stress-70 protein, HSC71, IDH, hnRNP C1/C2, and hnRNP F. In addition to 2-fold enhancement of apoptotic BFTC cells after 13-acetoxysarcocrassolide treatment displayed by flow cytometry, the regulation of specific proteins like HSP60 and hnRNPs F/H has been shown to be associated with pro-apoptotic elements including p53, caspases, involvement of mitochondria and Bcl-2 family [38,39,40]. Hence, we also investigated the changes of cytochrome C, p53, caspase-3, cleaved caspase-3, caspase-8, caspase-9, cleaved caspase-9 and Bcl-xL in the 13-acetoxysarcocrassolide-treated BFTC cells compared with the control. As a result, up-regulation of cytochrome C, p53, cleaved caspase-3 as well as cleaved caspase-9 and down-regulation of Bcl-xL after the treatment with 13-acetoxysarcocrassolide displayed by western blotting analysis further support the apoptosis-induced and anti-proliferative effects of 13-acetoxysarcocrassolide assessed by flow cytometry and MTT assay. 

Caspases are a group of intracellular cysteine proteases involved in the most essential pathways to induce apoptosis. Caspase-3, has been indicated to play an important role in nucleosomal DNA cleavage and promotion of the caspase activation [41]. Activation of caspase-3 led to apoptosis in breast cancer cells, bladder cancer cells, non-small cell lung cancer cells, neuroblastoma cells, prostate cancer cells, gastric cancer cells and cervical cancer cells [42,43,44,45,46,47]. Another member of the caspase family, caspase-9, has also been shown to be associated with the induction of apoptosis in several cancer cells including cervical cancer cells, human hepatoma cells, melanoma cells, and human lung cancer cells. Interestingly, the activation of caspase-3 is tightly linked to the activation of caspase-9 in these anti-tumor studies [48,49,50,51,52]. However, previous studies also showed that the apoptosis-induced activity exerted by the natural plant extract or chemical compound is correlated with caspase-3 and caspase-9, but not caspase-8 [49,51,52] In the current study, the increase in cleaved caspase-3 and cleaved caspase-9 was verified by western blot compared with the control as cleaved caspase-8 was not found in BFTC cells after exposure to 13-acetoxysarcocrassolide. These results support the apoptosis-induced effects of 13-acetoxysarcocrassolide on BFTC cells displayed by flow cytometry and are in accordance with the evidence on caspase-3 and caspase-9-associated apoptosis shown previously [48,49,50,51,52]. Moreover, these data demonstrate that activation of caspase-3 and caspase-9 is likely involved in induction of apoptosis by 13-acetoxysarcocrassolide in BFTC cells. 

Heat shock proteins are a group of housekeeping molecules and function as chaperones to recognize proteins with abnormal structures against stress conditions [53]. A member of this group, heat shock protein 60 (HSP60) has been found to widely exist in nature and act as chaperones to increase cell survival under physiological stress circumstances [54]. In this study, the expression of HSP60 was reduced in BFTC cells treated with 13-acetoxysarcocrassolide. Investigations previously indicated that HSP60 expression was up-regulated in cervical cancer tissue [55]. This protein is a contributor to the development of human cervical cancer and has been suggested as a biomarker for the disease [55]. It has been reported that HSP60 and other proteins like Ku70 binding protein, alpha enolase, and 26S proteasome subunit were up-regulated in the E7 oncogene in HPV-negative cervical cancer cell line (C33A), suggesting that the up-regulation of HSP60 by E7 oncogene could be one of the essential factors associated with resistance of apoptosis [56]. Studies indicated that the exposure of tumor suppressors such as mitomycin C and IGFBP7 to cervical and colorectal carcinoma cells caused down-regulation of HSP60. The down-regulation of HSP60 is suggested as being partially responsible for an inhibitory effect in colorectal cancer [57,58]. It has been found that HSP60 is able to promote the activation of pro-caspase-3 during apoptosis in earlier investigations [38]. Moreover, since the siRNA knockdown of HSP60 was shown to induce both mitochondrial and p53-dependent apoptosis, HSP60 inhibitors have been indicated as potential anti-cancer agents [59]. The correlation between drug resistance and the level of HSP60 in cancer cells has been studied. The basal levels of HSP60 were increased in cisplatin-resistant cervix squamous cell carcinoma cell subline A431/Pt than in non-resistant A431 cells [60]. In the current study, the down-regulation of HSP60 after treatment with 13-acetoxysarcocrassolide is consistent with previous studies [57,58,59,60]. Also, the increased expression of cleaved caspase-3, cytochrome C and p53 exhibited by western blot further connects the potential major role of HSP60 with induction of apoptosis in BFTC cells. Thus, these data suggest that the reduction of HSP60 is one of the important contributors to the anti-tumor effects of 13-acetoxysarcocrassolide and this is likely coupled with caspase-3, mitochondrial and p53-dependent apoptosis in BFTC cells.

Both stress-70 protein and heat shock cognate 71 kDa protein (HSC71), which play important roles in tumorigenesis and apoptosis of cancer cells, were up-regulated in the current study. Stress-70 protein acts as a chaperone involved in cell proliferation, differentiation, and tumorigenesis. The expression of Stress-70 protein greatly increased in pancreatic ductal carcinoma cells after treated with 5-aza-2′-deoxycytidine, a strong suppressor of the cancer cells [61]. The expression of stress-70 protein was also greatly elevated in erythroleukemia cells by radiation treatment. The over-expression of this protein was considered to be a relevant means of cell survival [62]. It has been reported that HSC71 is one of the most frequently found proteins in prostate cancer patients [63]. HSC71 was up-regulated in human neuroblastoma derived cells SH-SY5Y after treatment with staurosporine (STS), a broad spectrum protein kinase inhibitor and an extensively utilized apoptosis-inducer in the central nervous system [64]. In this study, the increased levels of stress-70 protein and HSC71 in BFTC cells treated with 13-acetoxysarcocrassolide suggest the association with the responses of either apoptosis or cell survival. 

Mutations of isocitrate dehydrogenase (IDH) gene have been identified in glioblastoma and were also investigated in thyroid cancer. Recently, the importance of IDH in anti-cancer therapeutics has been emphasized [65,66]. After administration with the chemotherapeutic agent from *Withania somnifera* along with paclitaxel, an effective chemical agent against lung cancer, significant up-regulation of the key enzymes in tricarboxylic acid (TCA) cycle such as IDH, succinate dehydrogenase, malate dehydrogenase, and alpha-ketoglutarate dehydrogenase was observed in lung cancer bearing animals, suggesting strong anti-tumor effects of the combination therapy [67]. Up-regulation of IDH1 has been correlated with the metastasis of human breast cancer [68]. Inhibition of IDH resulted in curcumin-induced apoptosis in the colon cancer cell line HCT116 [69]. Elevated expression of IDH in human esophageal squamous cell carcinoma has been reported [70]. A different alteration of this enzyme was reported in metastastic canine mammary carcinoma [71]. In this study, the down-regulation of IDH1 was observed by 2-DE analysis and verified by western blot analysis in BFTC cells treated with 13-acetoxysarcocrassolide. It suggested that the anti-cancer effects of 13-acetoxysarcocrassolide may be correlated with its effect on TCA cycle in BFTC cells and associated with the reduction of IDH.

The expressions of heterogeneous nuclear ribonucleoproteins (hnRNPs) in BFTC cells varied after treatment with 13-acetoxysarcocrassolide. It was found that hnRNP C1/C2 was greatly up-regulated, while hnRNP F was also up-regulated after the 13-acetoxysarcocrassolide treatment. HnRNP C1/C2 takes part in mRNA transcript packaging, splicing, nuclear retention, and mRNA stability. It was suggested that these proteins are associated with DNA-damage responses and radiation-induced apoptosis [72]. Reports indicated that hnRNP F was greatly expressed in a number of tumor cells except in hepatocellular carcinoma. In tumor tissues, highly elevated expression of hnRNP F was observed in both nuclei and cytoplasma although it was expressed higher in the nuclei than in the cytoplasma [73]. The different regulation between hnRNP F and hnRNP H has been found in gastric carcinoma and hepatocellular carcinoma [73]. The results of our study also showed different regulation of hnRNP F and hnRNP H in BFTC cells after treatment with 13-acetoxysarcocrassolide. It implicates that down-regulation of hnRNP H could be associated with decreased nuclei in BFTC cells resulting from apoptosis induced by the treatment. Reports indicate that hnRNP F is a biomarker for colorectal cancer [74]. Based on the results of this study, hnRNP F is also suggested as a biomarker for bladder tumors. It has been shown that hnRNPs F/H plays an essential role in the production of Bcl-xS, apoptosis-promoted proteins in the Bcl-2 family. The addition of hnRNP F increases the production of Bcl-xS and the knockdown of hnRNPs F/H by siRNAs decreases the ratio of Bcl-xS/BCL-xL [40]. The proteomic analysis and western blotting results of hnRNPs in this study showed up-regulation of both hnRNP C1/C2 and hnRNP F and down-regulation of hnRNP H while Bcl-xL expression was decreased after 13-acetoxysarcocrassolide treatment. These data suggest that the regulations of hnRNPs F/H and Bcl-xL were associated with the promotion of apoptosis induced by the treatment with 13-acetoxysarcocrassolide in BFTC cells. 

LDHB (l-lactate dehydrogenase B) is up-regulated after the 13-acetoxysarcocrassolide treatment. LDH release is an indicator of the integrity of cell membranes, since H_2_O_2_ treatment greatly elevates LDH level in normal cardiomyocytes [75]. The expression of LDHB was elevated in primary non-small cell lung cancer sera and progressively increased with increasing clinical stage. The level of LDHB also could be used to distinguish lung cancer from benign lung disease or healthy control groups with a sensitivity of 81%, a specificity of 70%, and a total accuracy of 76% [76]. It has been shown that the maintenance of low pyruvate level in colon cancer cells caused by the silencing of LDHB and the up-regulation of LDHA resulted in avoidance from cell death [77]. Increased expression of LDHB was also found in erythroleukemia cells treated with radiotherapy [62]. It is suggested that the up-regulation of LDHB in BFTC cells treated with 13-acetoxysarcocrassolide in the current study could be correlated with cytotoxic effects of 13-acetoxysarcocrassolide. 

The modulation of PDIA3 precursor and PPT1 in BFTC cells treated with 13-acetoxysarcocrassolide in the current study seems to present a paradigm shifting compared with some findings in previous reports. The functional roles of PDIA3 precursor in cells have been previously shown as a catalyst responsible for the rearrangement of disulphide bonds in the endoplasmic reticulum (ER) [78]. PDIA3 was shown as a potential biomarker in ovarian cancer and prostate cancer. It was further suggested that the reduction of caspase activity resulted from a decrease of PDIA3 in prostate cancer cell lines [79,80]. On the other hand, PPT1 is an enzyme which contributes to deacylation of palmitoylated proteins. Overexpression of PPT1 resulted in a 200-350% increase in depalmitoylating activity and was associated with the decrease in cell death and resistance to apoptosis induced by chemotherapeutic agents like C2-ceramide or Adriamycin. It has been suggested that protein palmitoylation could be a physiological regulator of apoptosis in human neuroblastoma cells [81,82]. In this study, 13-acetoxysarcocrassolide exhibited convincing evidence of anti-proliferation, anti-migration, and apoptosis-induction effects on BFTC cells *in vitro*. It is speculated that the down-regulation of PDIA3 and the up-regulation of PPT1 in BFTC cells treated with 13-acetoxysarcocrassolide, which were observed by 2-DE analysis and verified by western blot analysis, may not affect the cytotoxic effects of 13-acetoxysarcocrassolide on BFTC cells. Nevertheless, based on the results of study, PPT1 is a potential biomarker for bladder tumor.

## 5. Conclusion

In conclusion, combining the anti-proliferative, anti-migratory and apoptosis-induced results of 13-acetoxysarcocrassolide, we conclude that this cembranoid isolated from the Formosan soft coral exerted cytotoxic effects on bladder female transitional cancer (BFTC) cells *in vitro*. The regulation of critical proteins such as HSP60, HSC71, stress-70 protein, IDH, hnRNP C1/C2, hnRNP F, hnRNP H, and LDHB after 13-acetoxysarcocrassolide treatment, revealed by 2-DE, LC-MS/MS and western blot, suggested that these proteins are likely important biomarkers and may play major roles in the induction of apoptosis or the influence on energy metabolism in bladder transitional carcinoma. More noteworthy is that PPT1 and hnRNP F could be new biomarkers for bladder cancer. Furthermore, regarding modulation of HSP60 and hnRNPs F/H, up-regulation of cleaved caspase-3, cleaved caspase-9, cytochrome C, p53 as well as down-regulation of Bcl-xL, further demonstrates that the cytotoxic effects of 13-acetoxysarcocrassolide on BFTC cells are correlated with these pro-apoptotic factors.
